# Assessment of Blocking Interleukin-17 Antibodies in Tumor Immunotherapy with Checkpoint Inhibitors or Tumor-Specific T Cells in Implanted and UVB-Induced Cancers

**DOI:** 10.3390/biomedicines14081746

**Published:** 2026-08-03

**Authors:** Yuko Tsuruta, Carlos Alberto Mier-Aguilar, Sejong Bae, Nabiha Yusuf, Hui Xu

**Affiliations:** 1 Department of Dermatology, University of Alabama at Birmingham, Birmingham, AL 35294, USA; 2 General Internal Medicine & Population Science, University of Alabama at Birmingham, Birmingham, AL 35294, USA

**Keywords:** IL-17A blockade, tumor immunotherapy, PD-1 blockade, adoptive T-cell therapy

## Abstract

**Background:** Immune checkpoint inhibitors and adoptive T-cell therapies have substantially improved cancer treatment outcomes, but their use is often limited by immune-related toxicities, including cytokine release syndrome. The role of interleukin (IL)-17A in tumor immunity remains controversial, hindering its therapeutic applications in cancer. A concern is that application of IL-17 blocking agents to release cytokine storms caused by tumor immunotherapy reverses anti-tumor immunity. **Methods:** In this study, we evaluated the effect of IL-17A blockade alone and in combination with anti–PD (programmed cell death)-1 therapy or tumor-specific CD8^+^ T cells in melanoma, colon, and lung tumors, and in UVB-induced skin carcinogenesis. **Results:** Our results showed that blocking IL-17A inhibited tumor development, and did not impair the efficacy of tumor immunotherapies with checkpoint inhibitors or tumor-specific T cells. Combined treatment with anti-IL-17A and anti-PD-1 antibodies significantly enhanced tumor suppression compared to single-agent therapies in all tested tumor models. Moreover, IL-17A blockade improved the efficacy of adoptive CD8^+^ T-cell therapy. Mechanistic analyses revealed that the combination therapy increased infiltration of activated antigen-specific CD8^+^ T cells in tumors. In none of the tested tumor models did IL-17A blockade negatively affect the efficacy of tumor immunotherapy with checkpoint inhibitors or T-cell therapy. **Conclusions:** These findings suggest that the combined application of anti-IL-17A blocking agents with current tumor immunotherapy at the same time is a promising strategy to enhance the efficacy and potentially diminish immune-related adverse effects.

## 1. Introduction

Currently, both chimeric antigen receptor-modified T (CAR-T) cell therapy and immune checkpoint inhibitors, including anti-programmed cell death 1 (PD-1) and anti-programmed cell death 1 ligand 1 (PD-L1) antibodies, are widely used for standard and effective treatment in many types of cancer [1,2,3,4]. By inhibiting negative regulators of the immune system, those agents enhance immune hyperactivation against cancer cells, and aberrantly against host non-cancer cells, resulting in immune-related adverse events.

Cytokine release syndrome (CRS) is among the most serious adverse events of immune therapy and has been described as toxicities, including fatigue, headache, arthralgia, rash, pruritus, pneumonitis, diarrhea, colitis, hepatitis, and endocrinopathies [1,4,5]. To overcome CRS after CAR-T cell therapy and immune checkpoint inhibitors, anti-cytokine approaches, i.e., interleukin (IL)-6 receptor antibody (Ab) tocilizumab, have been reported [6,7].

Currently, different IL-17-blocking biologicals are used for the treatment of IL-17-mediated inflammatory diseases. Different side effects have been reported in patients during therapy.

Secukinumab, a human monoclonal Ab that selectively binds to circulating interleukin IL-17A, has been approved for the treatment of psoriasis and for treatment to overcome autoimmune-related flare-ups during immune checkpoint treatment [8]. Unfortunately, regardless of the patient’s recovery from autoimmune-related severe adverse effects, after the administration of IL-17A receptor inhibitors, such as secukinumab, it reversed the therapeutic effect of immune checkpoint therapy and tumors grew back. Thus, the application of IL-17A receptor inhibitors during tumor immunotherapy has been halted for nearly a decade.

The role of IL-17A cytokine is controversial in tumor development. IL-17 plays a role in promoting tumorigenesis, cancer progression, and metastasis through its unique signaling mechanism. Furthermore, IL-17-mediated innate inflammatory responses enhance resistance to cancer immunotherapy and chemotherapy [9]. On the other hand, some studies have reported a unique antitumor role of the IL-17 family in tumor progression by enhancing tumor surveillance and immunity [10]. Thus, re-evaluation of the role of IL-17A receptor inhibitors in cancer immunotherapy would provide new insights in the field of cancer immunotherapy as well as the possible adverse effects correlated with autoimmune diseases.

In the current study, we have found that blocking IL-17A cytokine enhances the efficacy of immunotherapy in implanted tumor models with immune checkpoint inhibitors and tumor-specific CD8 T cells. Mechanistic studies in the melanoma tumor model have shown that blocking both IL-17A and PD-1 results in increased infiltration of tumor-specific T cells in tumor tissue. These results suggest that tumor immunotherapy may be improved by adding anti-IL-17A antibodies.

## 2. Materials and Methods

### 2.1. Study Approval

The animal research protocol was approved by the Institutional Animal Care and Use Committee at the University of Alabama at Birmingham (#21428) and followed federal, state, and local guidelines.

### 2.2. Animals

C57BL/6J [Strain #000664] was purchased from the Jackson Laboratory (Bar Harbor, ME, USA). SKH1 Hairless Mouse [Strain #SKH1-*Hr^hr^*] was obtained from Charles River Laboratories (Wilmington, MA, USA). OT-1 mouse [Strain #003831, C57BL/6-Tg(TcraTcrb)1100Mjb/J] was originally obtained from the Jackson Laboratory (Bar Harbor, ME, USA). These mice contain transgenic inserts for mouse Tcra-V2 and Tcrb-V5 genes. The transgenic T cell receptor was designed to recognize ovalbumin peptide residues 257–264 (OVA_257–264_) in the context of H2K^b^ (CD8 co-receptor interaction with MHC class I).

### 2.3. Cell Lines

The OVA-transfected B16 tumor cell line (B16-OVA) was provided by Dr. Frances E. Lund (University of Alabama at Birmingham, Birmingham, AL, USA) and Richard W. Dutton (Trudeau Institute, Saranac Lake, NY, USA) [11]. Cells were maintained in RPMI 1640, supplemented with 10% heat-inactivated Fetal Bovine Serum, 2 mM of L-glutamine, 100 units/mL of penicillin, 100 μg/mL of streptomycin, 0.25 μg/mL of Amphotericin B, 0.05 mM of 2-Mercaptoethanol, 1 mM of sodium pyruvate, 100 μM of non-essential amino acids, and 400 μg/mL of G418 (Thermofisher Scientific, Waltham, MA, USA). MC-38 colon carcinoma cell line was obtained from Kerafast, Inc. (Shirley, MA, USA) and maintained according to the manufacturer’s instructions. LL/2 (LLC1) [CRL-1642^™^] Lewis lung carcinoma cell line was obtained from American Type Culture Collection (ATCC, Manassas, VA, USA) and maintained according to the manufacturer’s instructions.

### 2.4. Combination Targeted and Immune-Based Therapy in an Immunogenic Mouse Model

For the B16-OVA melanoma model, we intradermally injected female C57BL/6J mice (9–11 weeks of age) with 1 × 10^5^ B16-OVA melanoma cells resuspended in 50 μL PBS mixed with 50 μL Matrigel^®^ (Corning, Tewksbury, MA, USA). For the MC-38 colon cancer model, we injected (intradermally) female C57BL/6J mice (9 weeks of age) with a cell suspension containing 1 × 10^6^ MC-38 cells resuspended in 100 μL PBS. For the Lewis lung carcinoma model, we intradermally injected female C57BL/6J mice (13 weeks of age) with 0.5 × 10^6^ LL/2 cells resuspended in 100 μL PBS. Intraperitoneal injection of treatment antibodies started on the same day of the intradermal injection or seven days after the intradermal injection, every three days until termination. Treatment antibodies (Abs) were 100 μg of isotype control (InVivoMAb mouse IgG1 isotype control, Clone: MOPC-21, BioXCell, Lebanon, NH, USA), 100 μg of anti-mouse IL-17A (IL-17A Ab) (InVivoMAb anti-mouse IL-17A, Clone:17F3, BioXCell), 200 μg of anti-mouse PD-1 (PD-1 Ab) (InVivoMAb anti-mouse PD-1 (CD279), Clone RMP1–14, BioXCell), or the combination of 100 μg of IL-17A Ab and 200 μg of PD-1 Ab. The tumor grew and mice were monitored daily for survival.

### 2.5. UVB Source

A simulated solar ultraviolet (UV) source system [UVA/UVB Research Irradiation Unit] (Daavlin, Bryan, OH, USA) was outfitted with a bank of four UVB lamps and an electronic controller to regulate UVB dosage at a fixed distance of 24 cm between the lamps and the dorsal skin surface of mice. Using Kodacel cellulose film, wavelengths less than 290 nm were filtered out (Eastman Kodak Co., Rochester, NY, USA). Most of the resulting wavelengths were in the UVB (290–320 nm; 80%) and UVA (20%) ranges, with peak emission at 314 nm, as monitored regularly.

### 2.6. Combination Targeted and Immune-Based Therapy in UVB-Induced Photocarcinogenesis Model

The dorsal skin of panels of SKH1 hairless mice was exposed to a single dose of UVB radiation (180 mJ/cm^2^), three times a week over the course of photocarcinogenesis experiments. Mice were monitored for tumors on a weekly basis. After 100% tumor incidence, all mice were randomly divided into four experimental groups. Grouped mice were intraperitoneally injected with 100 μg of isotype control, 100 μg of IL-17A Ab alone, 200 μg of PD-1 Ab alone, or the combination of 100 μg of IL-17A Ab and 200 μg of PD-1 Ab weekly. Tumor growth in mice was monitored weekly until termination according to IACUC guidelines.

### 2.7. Preparation and Generation of OVA-Specific CD8 T Cells

Spleens and lymph nodes were collected from male OT-1 mice (11 weeks of age) and single-cell suspensions were separated by mechanical disruption. After Ammonium-Chloride-Potassium Lysing Buffer (Gibco, Grand Island, NY, USA) treatment of cells for the lysis of red blood cells in samples, cells underwent magnetic-activated cell sorting (MACS) by CD8a (LY-2) MicroBeads positive selection according to the manufacturer’s protocol (Miltenyi Biotec, San Diego, CA, USA). Enriched CD8-positive OVA-specific T cells were resuspended in PBS for intravenous injection.

### 2.8. Combination Targeted and Adoptive T Cell Immunotherapy in the Immunogenic Mouse Model

Female C57BL/6J mice (11 weeks of age) received an intradermal injection containing 1 × 10^5^ B16-OVA melanoma cells resuspended in 50 μL PBS mixed with 50 μL Matrigel^®^ (Corning, Tewksbury, MA, USA) on day 0. After seven days of tumor cell inoculation, we randomly divided all mice into four experimental groups. We then enriched CD8-positive OVA-specific T cells and injected them into tumor-bearing mice via the tail vein with or without intraperitoneal injection of 100 μg of IL-17A Ab. IL-17A Ab was injected every three days until the end of the experiment. Tumor growth was monitored daily.

### 2.9. Tumor Cell Preparation

We digested the tumors using the GentleMACS^TM^ Octo Dissociator [Pre-installed Program 37C_M_TDK_1, at 37 C for 41 m] (Miltenyi Biotec, San Diego, CA, USA) with 2.7 mg/mL Collagenase from *Clostridium histolyticum*, (Sigma-Aldrich, Inc., St. Louis, MO, USA), 0.25 mg/mL Hyaluronidase from bovine testes (Sigma-Aldrich, Inc., St. Louis, MO, USA), 0.1 mg/mL Deoxyribonuclease I from bovine pancreas lyophilized powder (Sigma-Aldrich, Inc., St. Louis, MO, USA), and 10 mM HEPES (Gibco, Grand Island, NY, USA) in RPMI 1640, supplemented with 10% heat-inactivated Fetal Bovine Serum. Single-cell suspensions were obtained through 70 μm pore Falcon™ Cell Strainers (Corning, Glendale, AZ, USA).

### 2.10. Lymphocyte Preparation

The axillary and inguinal draining lymph nodes were harvested and crushed into small pieces. The cell suspensions were filtered through a 70 μm pore Falcon™ Cell Strainer to remove debris, and single-cell suspensions were collected for downstream flow cytometry analysis.

### 2.11. Antibodies and Flow Cytometry

For the phenotype analysis of tumor cells and lymphocytes, single-cell suspensions were stained with fluorochrome-conjugated antibodies with specificity to mouse cell surface antigens in the presence of Fc receptor blocking with InVivoPlus anti-mouse CD16/CD32 (clone 2.4G2, BioXCell) before acquisition in a flow cytometer. The following antibodies were used: anti-CD4 (clone RM4–5, Super Bright™ 436, eBioscience™ Invitrogen, Carlsbad, CA, USA)**,** anti-CD8a (clone 53–6.7, BV650, BD, San Diego, CA, USA), anti-CD45 (clone 30-F11, PE/Cyanine5, BioLegend, San Diego, CA, USA), anti-CD44 (clone IM7, PE-CF594, BD), anti-CD279 (PD-1) (clone J43, PE-Cyanine7, eBioscience™ Invitrogen), anti-CD366 (TIM-3) (clone 5D12/TIM-3, R718, BD), anti-CD223 (LAG-3) (clone C9B7W, APC/Fire™ 750), anti-CD49b (clone HMα2, Alexa Fluor^®^ 488 (BioLegend, San Diego, CA, USA), and iTAg Tetramer/PE—H-2 Kb OVA (SIINFEKL) (MBL International, Woburn, MA, USA).

For the intracellular cytokine analysis, BD Cytofix/Cytoperm™ Plus Fixation/Permeabilization Solution Kit with BD GolgiPlug™ (BD Biosciences, Milpitas, CA, USA) was applied according to the manufacturer’s instructions. Briefly, single cell suspensions were stimulated for 4 h with PMA 50 ng/mL, Ionomycin 500 μg/mL, and GolgiPlug™ (BD) for cell permeabilization in the RPMI 1640 supplemented with 10% heat-inactivated Fetal Bovine Serum, 2 mM of L-glutamine, 100 units/mL of penicillin, 100 μg/mL of streptomycin, 0.25 μg/mL of Amphotericin B, 0.05 mM of 2-Mercaptoethanol, 1 mM of sodium pyruvate, 100 μM of non-essential amino acids, and 10 mM HEPES (Gibco). Then, cells were resuspended in staining media with antibodies (anti-IFN-γ: clone XMG1.2, PE-Cy7, BD or anti-IL-17A: clone TC11–18H10, Alexa Fluor™ 700, BD) for the detection of intracellular cytokines.

LIVE/DEAD™ Fixable Aqua Dead Cell Stain Kit for 405 nm excitation (Invitrogen, Carlsbad, CA, USA) was used to exclude dead cells. The stained cells were fixed with 4% paraformaldehyde, acquired on a CytoFLEX S Flow Cytometer (BECKMAN COULTER, Indianapolis, IN, USA), and analyzed with FLOWJO 10.9.0 (BD, Ashland, OR, USA).

### 2.12. Analysis of Tumor Cells and Lymphocytes Using Uniform Manifold Approximation and Projection for Dimension Reduction (UMAP) and the Leiden Algorithm for Cluster Identification

Before UMAP analysis and cluster identification of T cell subsets, we curated Flow Cytometry Standard (FCS) files in FLOWJO 10.9.0 by removing outliers using the Peak Extraction And Cleaning Oriented Quality Control (PeacoQC) algorithm downloaded from the FLOWJO Exchange. Where needed, we adjusted the compensation parameters to improve visualization and clustering before PeacoQC processing.

In the curated FCS files, we performed regular gating and axis adjustments for our parameters and selected the CD4+ or CD8+ cells. We then exported these cells as new FCS files and performed down-sampling using the opdisDownsampling and flowCore packages within R [12,13]. We obtained an identical number of cells for all samples to avoid uneven sample contributions for the final analysis. FCS files with down-sampled cells (300 cells for tumor and 10,000 cells for lymphocytes) were imported into FLOWJO 10.9.0 and numerical identifiers corresponding to treatment and sample ID were assigned individually. After this, we concatenated all FCS files into a new FCS file. We then exported the scaled values of all fluorochrome parameters, forward scatter, side scatter, treatment, and sample ID from a concatenated new FCS file into a CSV file.

Using a newly generated CSV file, we performed UMAP analysis in Python (Python v3.11), using the UMAP package [14]. Concurrently, we used the Leidenalg function from the SCANPY package to perform cluster-ID generation, applying the Leiden algorithm [15,16]. After testing different resolution parameters for optimal identification of cluster numbers, data were exported into a new CSV file. Showing each parameter expression level, a bar plot was adapted from FlowKit, a Gating-ML 2.0-compliant Python package for cluster comparison [17]. Applying a new CSV file, visualization of the clusters and UMAP plots was presented in FLOWJO 10.9.0. In R, a newly generated CSV file was used for the analysis among treatment groups. Further validation of protein expression was performed using FLOWJO 10.9.0.

### 2.13. Statistical Analysis

Tumor volume was calculated by using the following formula: tumor volume (mm^3^) = (length) × (width) × (height) × 0.5 [18]. Differences among groups were analyzed for statistical significance by one-way ANOVA followed by post hoc Tukey’s test using GraphPad Prism 10.1.0 (GraphPad Software, Inc., La Jolla, CA, USA) and R software (v 4.3.1). *p* < 0.05 was considered statistically significant. The results were expressed as mean plus/minus the standard error of the mean (SEM).

## 3. Results

### 3.1. The Combination of Anti-IL-17 with Anti-PD-1 Antibodies Benefits the Efficacy of Tumor Immunotherapy Across Different Tumor Types

**Figure 1:**
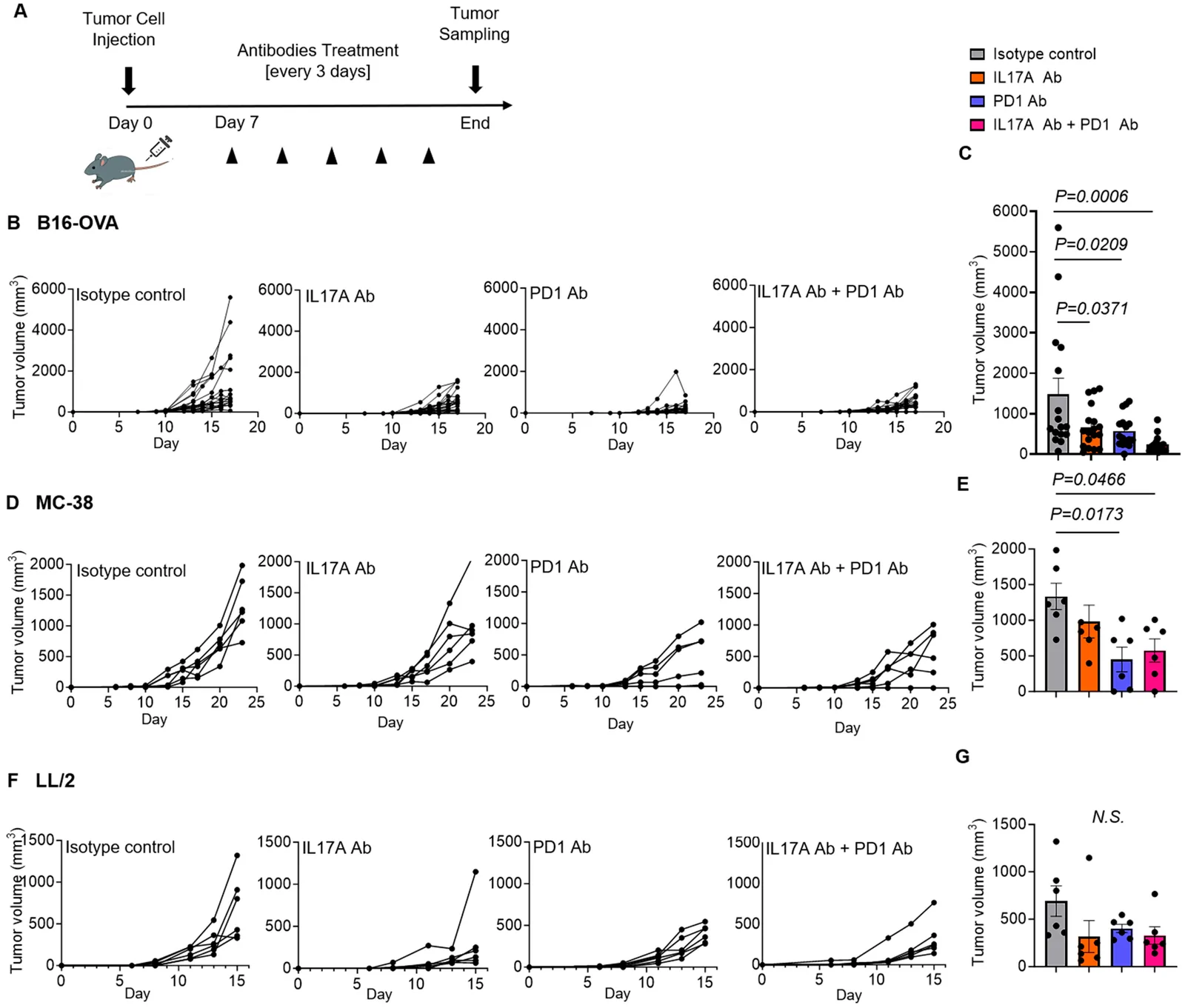
Tumor growth was suppressed after treatment with the combination of anti-mouse IL-17A (IL-17A Ab) and anti-mouse PD1 (PD1 Ab) in C57BL/6J mice. (**A**) Schematic overview of the protocol used to assess the impact of antibody therapy on tumor-inoculated C57BL/6J mice. C57BL/6J mice (female, 10–13 weeks, the Jackson Laboratory) were intraperitoneally injected with 100 μg mouse IgG1 isotype control (isotype control) or 100 μg of IL-17A Ab or 200 μg of PD1 Ab or the combination of 100 μg of IL-17A Ab and 200 μg of PD1 Ab on day 7 after tumor implantation on the back subcutaneously. Antibody injection continued every three days before termination. (**B**) Tumor growth over time of B16-OVA tumor-bearing mice treated with antibodies intraperitoneally. Serial tumor measurements were obtained. Values for individual mice are plotted. n = 18 mice per group. (**C**) Tumor volume at day 17 with B16-OVA tumor-bearing mice treated with antibodies intraperitoneally. Data are represented as mean values. Error bars show the standard error of the mean. *p*-values represent statistical comparisons between the two groups using one-way ANOVA followed by post hoc Tukey’s test, which was used to determine significance among groups. (**D**) Tumor growth over time of MC-38 Cell Line (MC-38) tumor-bearing mice treated with antibodies intraperitoneally. Serial tumor measurements were obtained. Values for individual mice are plotted. n = 6 mice per group. (**E**) Tumor volume at day 23 with MC-38 tumor-bearing mice treated with antibodies intraperitoneally. Data are represented as mean values. Error bars show the standard error of the mean. *p*-values represent statistical comparisons between the two groups using one-way ANOVA followed by post hoc Tukey’s test, which was used to determine significance among groups. (**F**) Tumor growth over time of LL/2 (LLC1) Lewis lung carcinoma (LL/2) tumor-bearing mice treated with antibodies intraperitoneally. Serial tumor measurements were obtained. Values for individual mice are plotted. n = 6 mice per group. (**G**) Tumor volume at day 15 with LL/2 tumor-bearing mice treated with antibodies intraperitoneally. Data are represented as mean values. Error bars show the standard error of the mean. One-way ANOVA followed by post hoc Tukey’s test was used to determine statistical significance.

We studied the effect of a combination of anti-IL-17A and anti-PD-1 antibodies for immunotherapy in three mouse tumor models. After seven days of tumor inoculation on the back of C57BL/6J mice, they were treated with antibodies intraperitoneally every three days (Figure 1A). In the mouse melanoma model with B16-OVA cell lines, the combination therapy group with IL-17A Ab and PD-1 Ab achieved a significant reduction in tumor growth compared with the isotype control group (*p* = 0.0006) on day 17 (Figure 1B,C). Treatment with only IL-17A or only PD-1 abs resulted in tumor reduction compared with the isotype control group (*p* = 0.0209 and *p* = 0.0371, respectively) on day 17 (Figure 1B,C). Similarly, in the MC-38 mouse colon carcinoma model, tumor growth was significantly suppressed in the combination therapy group (*p* = 0.0466) as well as the PD-1 Ab group (*p* = 0.0173) compared with the isotype control group (Figure 1D,E). Although the combination therapy and the mono-Ab therapy did not show a significant outcome in the mouse lung carcinoma model with LL/2 cell lines, the combination therapy and the mono IL-17A Ab therapy were significantly effective compared with the isotype control group (*t*-test, *p* = 0.0411 and *p* = 0.0411, respectively) (Figure 1F,G). The combination therapy was predominantly effective in all three tumor models, regardless of cell types. Interestingly, IL-17A Ab therapy alone was effective and harmless in all three tumor models as well.

### 3.2. The Combination of Anti-IL-17 with Anti-PD-1 Antibodies Enhances the Prevention of UVB-Induced Skin Carcinogenesis

**Figure 2:**
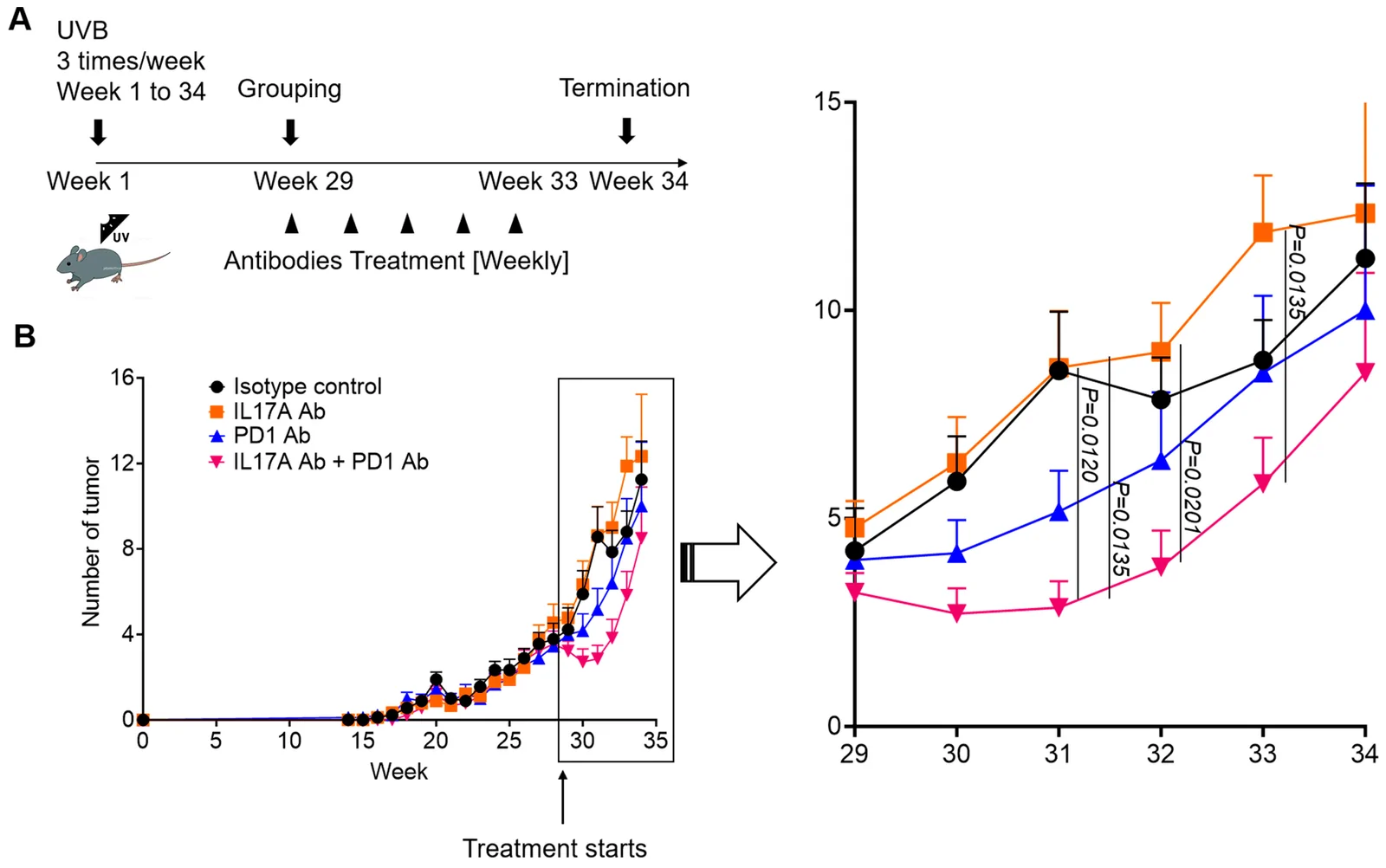
UVB-induced skin carcinogenesis in SKH1 hairless mice was reduced after treatment with the combination of IL-17A Ab and PD1 Ab in SKH1 hairless mice. (**A**) Schematic overview of the protocol used to assess the impact of antibody therapy on UVB-induced skin carcinogenesis in SKH1 hairless mice. SKH1 hairless mice were irradiated with UVB (180 mJ/cm^2^) three times per week, continuing for 34 weeks (male n = 24, female n = 21, starting at 8–15 weeks, The Charles River Laboratories). Distinguishable tumors (initially arising as papules) were counted and measured weekly. At 29 weeks with 100% tumor incidence, thirty-eight mice were randomly divided into 4 experimental groups. Grouped mice were intraperitoneally injected with 100 μg isotype control or 100 μg of IL-17A Ab or 200 μg of PD1 Ab or the combination of 100 μg of IL-17A Ab and 200 μg of PD1 Ab weekly between 29 and 33 weeks. All mice were terminated at week 34 according to IACUC guidelines. (**B**) Number of tumors over time in SKH1 hairless mice treated with antibodies intraperitoneally. n = 4 or 5 mice per group. The average tumor number per group was plotted. Error bars show the standard error of the mean. One-way ANOVA followed by post hoc Tukey’s test was used to determine statistical significance.

To further prove the effectiveness of combination targeted and immunotherapy with IL-17A Ab and PD-1 Ab, we utilized the UVB-induced carcinogenesis model in SKH1 mice. As shown in the schematic overview (Figure 2A), we irradiated SKH-1 mice with UVB three times a week for the entire 34 weeks of the experimental period. In week 29, 100% tumor incidence was confirmed, and mice were randomized into four treatment groups. Weekly antibody therapy was conducted until termination. As shown in Figure 2B, the combination therapy group with IL-17A Ab and PD-1 Ab showed a significantly smaller number of tumors compared with the isotype control group (*p* = 0.0120 at week 31) and the IL-17A Ab group (*p* = 0.0135 at week 31, *p* = 0.0201 at week 32, *p* = 0.0135 at week 33). Statistical analysis with the t-test indicated that the combination therapy group had lower tumor numbers at week 30 (*p* = 0.0220) and week 32 (*p* = 0.0128) than the isotype control group. The carcinogenesis model by SKH1 mice confirmed the advantage of the combination therapy.

### 3.3. The Addition of Anti-IL-17 Antibodies Enhances T Cell Therapy for Melanoma

**Figure 3:**
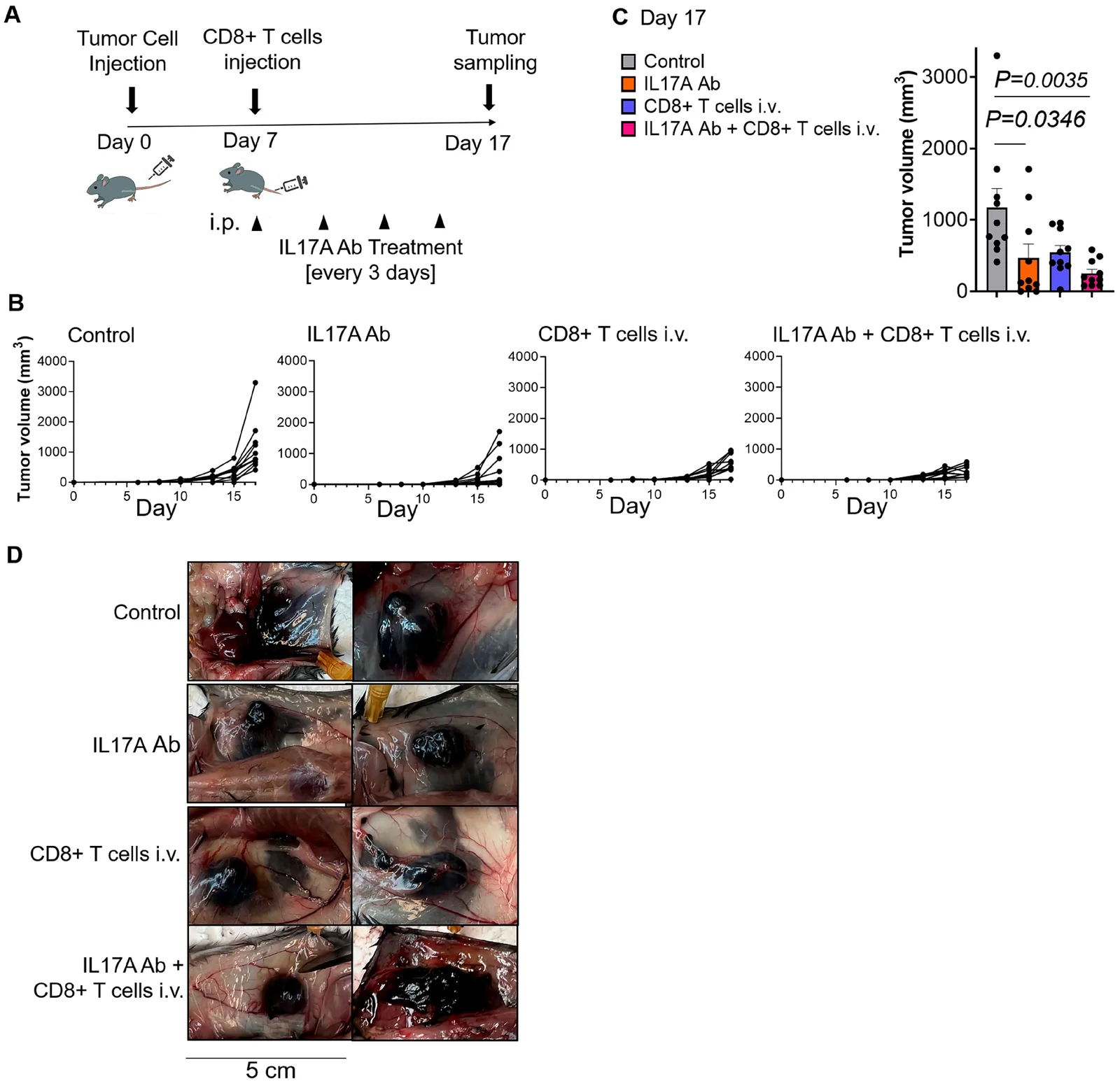
IL-17A Ab treatment enhanced tumor regression with OVA-specific effector T cells purified from OT-1 mice (OT-1 T cells) in B16-OVA tumor-bearing C57BL/6J mice. (**A**) Schematic overview of the protocol used to assess the impact of IL-17A Ab treatment with OT-1 T cells on tumor-inoculated C57BL/6J mice. C57BL/6J mice (female, 11 weeks, n = 40, The Jackson Laboratory) were implanted subcutaneously on the back with B16-OVA cells on day 0. OT-1 T cells were administered intravenously to mice before the appearance of established subcutaneous tumors on day 7. On the same day, 100 μg of IL-17A Ab was administered intraperitoneally. IL-17A Ab injection continued every three days until termination on day 17. (**B**) Tumor growth over time of B16-OVA tumor-bearing mice treated with IL-17A Ab intraperitoneally and/or OT-1 T cells intravenously, along with a no-treatment group as a control. Serial tumor measurements were obtained. Values for individual mice are plotted. n = 10 mice per group. (**C**) Tumor volume on day 17 with B16-OVA tumor-bearing mice treated with IL-17A Ab intraperitoneally and/or OT-1 T cells intravenously along with a no-treatment group as a control. Data are represented as mean values. Error bars show the standard error of the mean. One-way ANOVA followed by post hoc Tukey’s test was used to determine statistical significance. (**D**) Representative images on day 17 are shown along with observed subcutaneous tumors under the skin for the treatment group of IL-17A Ab intraperitoneally and/or OT-1 T cells intravenously, along with the no-treatment group as a control.

To investigate the impact of IL-17 pathway blockage in immune therapy settings, we further introduced the model of adoptive T cell immunotherapy. We assessed the therapeutic efficacy of IL-17A Ab treatment combined with applied OVA-specific effector T cells in a B16-OVA tumor-bearing model. As shown in Figure 3A, IL-17A Ab (every three days) and/or OVA-specific effector T cells purified from OT-1 mice (OT-1 T cells) (once) were injected seven days after inoculation of B16-OVA cell lines into the backs of C57BL/6J mice. Compared with the non-treatment group, tumor growth was significantly reduced in the IL-17A Ab group (*p* = 0.0346) and the group receiving the combination of IL-17A Ab and OT-1 T cells (*p* = 0.0035) (Figure 3B,C). In Figure 3D, representative images of subcutaneous B16-OVA tumors are presented, showing the tumor size reduction with IL-17A Ab and/or OT-1 CD8 treatment compared with the non-treatment group on day 17. Here, the adoptive T cell immunotherapy was enhanced with IL-17 pathway blockade in the murine melanoma model.

### 3.4. The Addition of Anti-IL-17 Antibodies Increases the Infiltration of Antigen-Specific CD8 T Cells in Tumors

**Figure 4:**
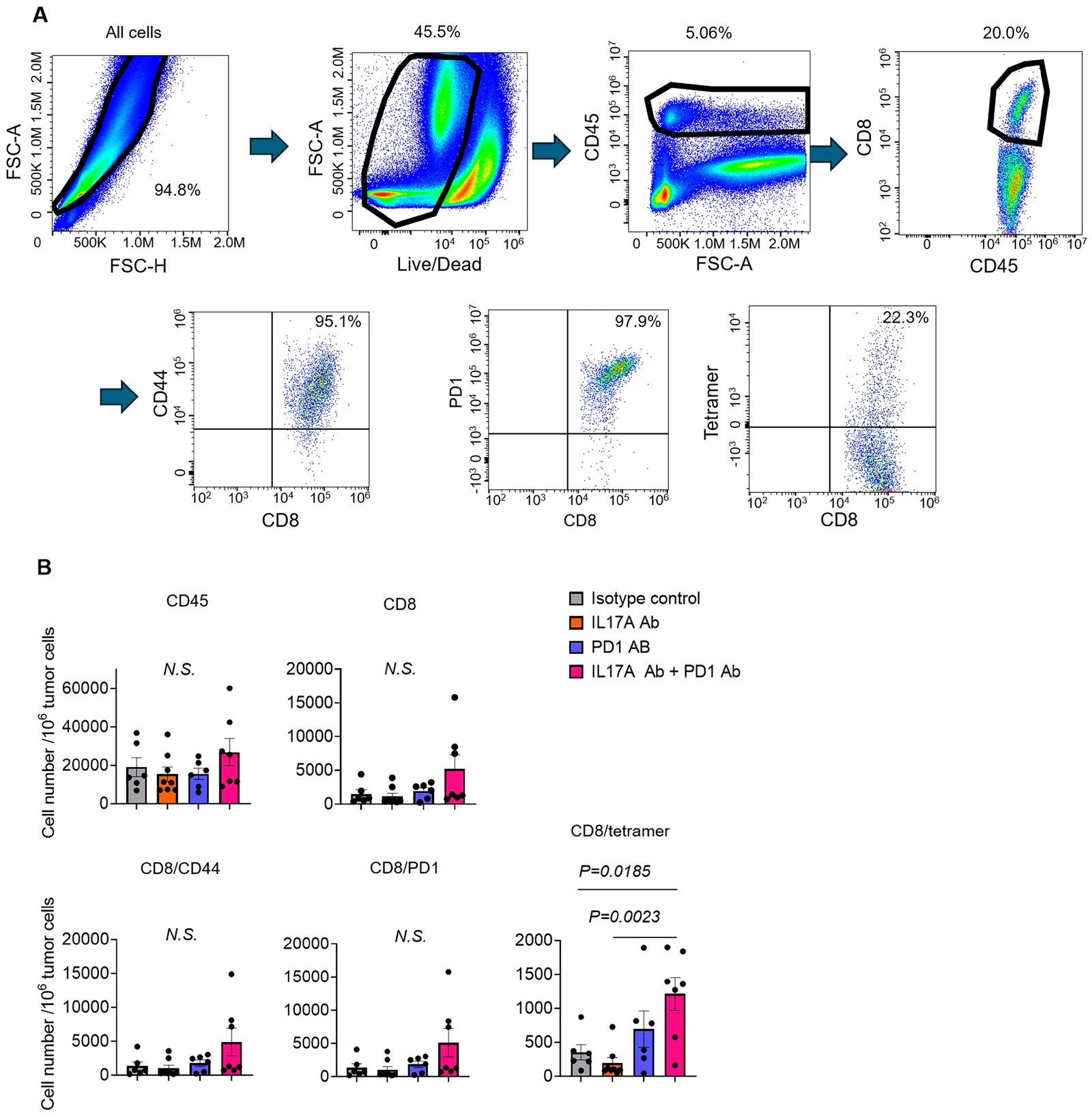
The combination treatment with anti-mouse IL-17A (IL-17A Ab) and anti-mouse PD1 (PD1 Ab) enhanced infiltration of cytotoxic T cells into B16-OVA tumors in C57BL/6J mice. Single cells were prepared from the tumor samples, which were collected after antibody therapy on tumor-inoculated C57BL/6J mice (supplemental figure for experimental design and tumor growth outcome). (**A**) Gating strategy for flow cytometry analysis. Single tumor cells were selected in a forward scatter-height vs. forward scatter-area dot plot, and live tumor cells were subsequently selected in a live/dead vs. forward scatter-area dot plot. Then, leukocytes in the tumor were selected in a forward scatter-area vs. CD45 dot plot. After the selection of leukocytes, CD8+ lymphocytes were identified in a CD45 vs. CD8 dot plot. Furthermore, among CD8+ lymphocytes, three important populations were gated. CD8+CD44+ lymphocytes were gated as effector/memory CD8 T cells in a CD8 vs. CD44 dot plot. CD8+PD1+ lymphocytes were gated in a CD8 vs. PD1 dot plot. CD8+Tetramer+ lymphocytes were gated as activated antigen-specific CD8 T cells in a CD8 vs. tetramer dot plot. (**B**) The bar graphs depict the mean of cell numbers per one million live tumor cells per group. Error bars show the standard error of the mean. One-way ANOVA followed by post hoc Tukey’s test was used to determine statistical significance. Cell counts are for CD45+ cells, CD45+CD8+ cells, CD45+CD8+CD44+ cells, CD45+CD8+PD1+ cells, and CD45+CD8+tetramer+ cells.

To examine mechanisms underlying the combined immunotherapy with IL-17A Ab and PD-1 Ab, we analyzed B16-OVA tumor tissues. C57BL/6J mice (female, 7–8 weeks, Jackson Laboratories, Bar Harbor, ME, USA) were intraperitoneally injected with 100 μg mouse IgG1 isotype control (isotype control) or 100 μg of IL-17A Ab or 200 μg of PD-1 Ab or the combination of 100 μg of IL-17A Ab and 200 μg of PD-1 Ab immediately after B16-OVA cell line inoculation. Single tumor cells were prepared from the tumor samples for flow cytometry analysis for CD4, CD8, CD45, CD44, PD-1, and iTAg tetramer. After gating CD45+CD8+ T lymphocytes, fractions of those cells were further separated into subpopulations of CD8+CD44+, CD8+PD-1+, and CD8+tetramer+ (Figure 4A).

The combination treatment with IL-17A Ab and PD-1 Ab enhanced infiltration of CD45+CD8 T cells into the B16-OVA tumor in C57BL/6J mice compared with solely IL-17A Ab treatment (*p* = 0.0205 with *t*-test) (Figure 4B). An increased infiltration of effector T cells in the tumor was observed by the CD8+/CD44+ subpopulation compared with IL-17A Ab treatment (*p* = 0.0205 with *t*-test). This was supported by the enhanced OVA-specific T cell tumor infiltration analyzed by the CD8+tetramer+ subpopulation in the combination treatment (*p* = 0.0185 vs. isotype control and *p* = 0.0023 vs. IL-17A Ab treatment). Interestingly, in the CD8+PD-1+ subpopulation, exhausted T cells have shown an increase in the combination treatment compared with solely IL-17A Ab treatment (*p* = 0.0205 with *t*-test).

### 3.5. The Combination of IL-17A Ab and PD-1 Ab Induced Infiltration of Effector CD8+ T Cells and OVA-Specific CD8+ T Cells in the Tumor

**Figure 5:**
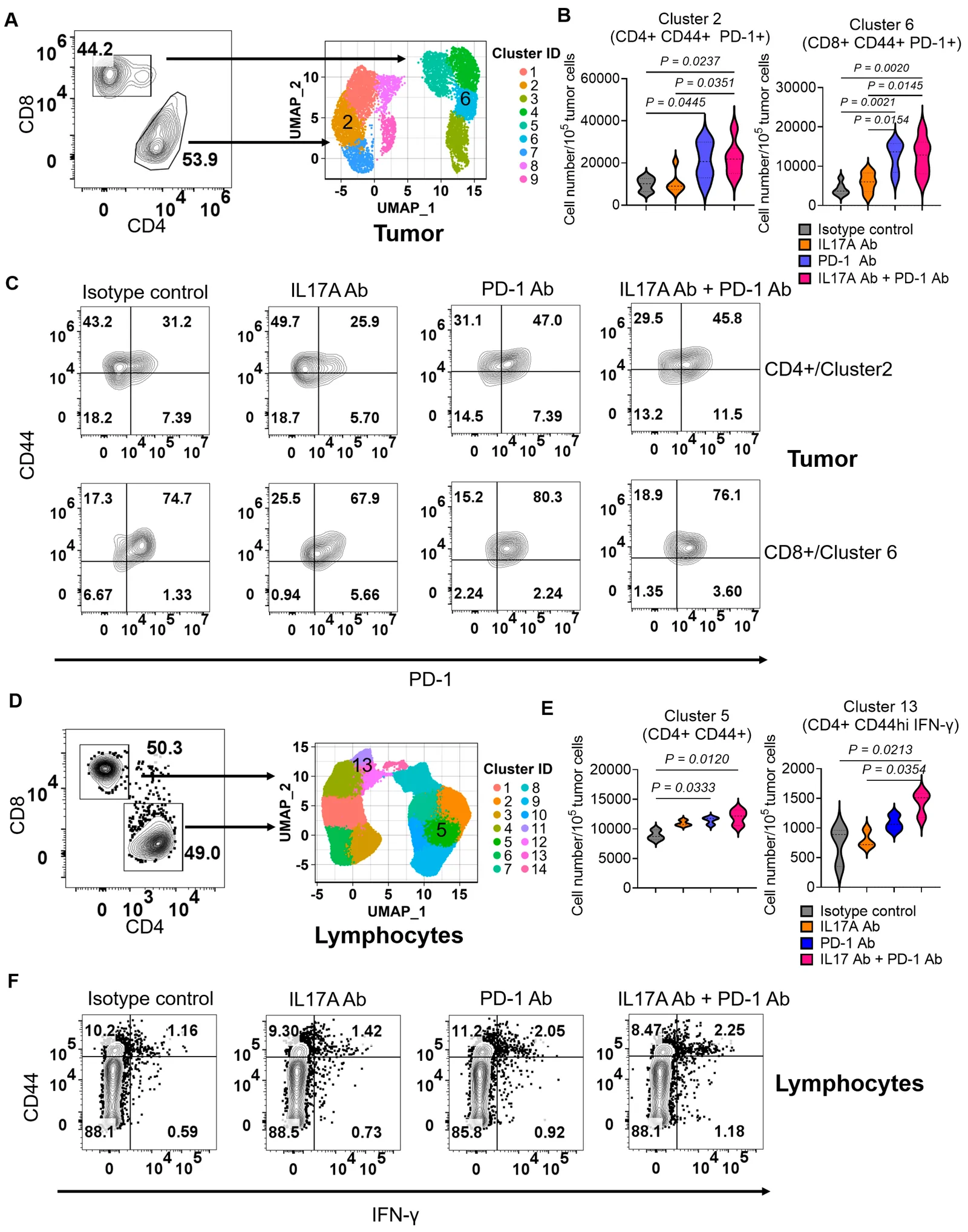
Activated CD4+ and CD8+ cells are increased in both tumors and draining lymph nodes of mice treated with combination therapy of anti-mouse IL-17A (IL-17A Ab) and anti-mouse PD-1 (PD-1 Ab). C57BL/6J mice (female, 10 weeks, The Jackson Laboratory) were intraperitoneally injected with 100 μg mouse IgG1 isotype control (isotype control) or 100 μg of IL-17A Ab or 200 μg of PD-1 Ab or the combination of 100 μg of IL-17A Ab and 200 μg of PD-1 Ab on day 7 after tumor implantation on the back subcutaneously. Antibody injection continued every three days before termination. Single tumor cells from the tumor samples and lymph nodes on day 17 were prepared for flow cytometry analysis with the following antibodies: anti-CD4, anti-CD8a, anti-CD44, anti-CD279 (PD-1), anti-CD366 (TIM-3), anti-CD223 (LAG-3), and anti-CD49b [tumor and lymph nodes], iTAg Tetramer [tumor], IFN-γ and IL-17 [draining lymph nodes]. (**A**) Analysis of tumor-infiltrated CD4+ and CD8+ T cells using UMAP identified nine clusters based on the expression of surface markers. (**B**) Unsupervised quantitative comparison among each treatment group for Cluster 2 (CD4+ CD44+ PD-1+) and Cluster 6 (CD8+ CD44high PD-1+). Six mice per treatment group were analyzed. One-way ANOVA with post hoc Tukey’s test was used to determine statistical significance. (**C**) Conventional flow analysis for CD44 and PD-1 expression in Cluster 2 and Cluster 6 for each treatment group. (**D**) Analysis of CD4+ and CD8+ T cells from draining lymph nodes using UMAP identified 14 clusters based on the expression of different surface markers and cytokines. (**E**) Unsupervised quantitative comparison among each treatment group for Cluster 5 (CD4+ CD44+) and Cluster 13 (CD8+ CD44high IFN-γ+). Three mice per treatment group were analyzed. One-way ANOVA with post hoc Tukey’s test was used to determine statistical significance. (**F**) Conventional flow analysis for CD44 and IFN-γ expression in lymphocytes for each treatment group.

In further analysis of T cells in tumor samples and draining lymph nodes, anti- CD366 (TIM-3), anti-CD223 (LAG-3), anti-CD49b, and iTAg Tetramer were applied. Using UMAP in combination with the Leiden algorithm for cluster identification, we analyzed CD4+ and CD8+ T cells (Figure 5A). Out of nine clusters identified, two clusters, cluster 2 and cluster 6, were significantly increased in the combination therapy group with IL-17A Ab and PD-1 Ab compared to the isotype control group (*p* = 0.0237 and *p* = 0.0020, respectively) (Figure 5B). Analysis of the cell markers expressed in these clusters has revealed that cluster 2 corresponds to CD4+ CD44+ PD-1+ cells, while cluster 8 corresponds to CD8+ CD44+ PD-1+ cells. Further analysis of these clusters using conventional flow cytometry corroborated these findings (Figure 5C). These results suggest that the combination therapy group with IL-17A Ab and PD-1 Ab may result in an increased number of activated (both CD4 and CD8) infiltration in the tumors, compared with the no treatment group [insert reference about CD44 PD1 activated cells after PD1 treatment].

Considering that T cell responses require activation of T cells in secondary lymphoid organs, we analyzed the draining lymph nodes of these mice on the same day of tumor analysis (day 17). Single-cell suspensions of lymph nodes were prepared for flow cytometry analysis with the following antibodies: anti-CD4, anti-CD8a, anti-CD44, anti-CD279 (PD-1), anti- CD366 (TIM-3), anti-CD223 (LAG-3), anti-CD49b, anti-IFN-γ, and anti-IL-17A. CD4+ and CD8+ T cells were analyzed with UMAP in combination with the Leiden algorithm for cluster identification (Figure 5D). Out of 14 clusters identified, two clusters, cluster 5 and cluster 13, were significantly increased in the combination therapy group with IL-17A Ab and PD-1 Ab compared to the isotype control group (*p* = 0.0120 and *p* = 0.0213, respectively) (Figure 5E). Analysis of the cell markers expressed in these clusters has revealed that cluster 5 corresponds to CD4+ CD44+ cells, while cluster 13 corresponds to CD8+ CD44high IFN-γ+ cells. Further analysis of these clusters using conventional flow cytometry corroborated these findings (Figure 5F). These results suggest that the combination therapy group with IL-17A Ab and PD-1 Ab results in an increased number of activated CD4+ or CD8+ T cells in draining lymph nodes, compared with the no treatment group.

## 4. Discussion

Interleukin 17 is a highly versatile proinflammatory cytokine necessary for vital processes, including host immune defenses, tissue repair, inflammatory disease pathogenesis, and cancer progression. However, the role of IL-17 signaling in tumor development remains a complex subject, and conflicting results have been reported in the last decade, since both pro- and anti-tumor effects have been described [9,19,20]. Studies that evaluated the association between IL-17 and patients’ prognoses are inconsistent across cancer types, including melanoma. In the murine colon cancer model, the anti-tumor effect of *IL-17* gene transfection was presented by increased infiltration of lymphocytes in tumor tissues and high expression of interferon-gamma (IFNγ) in tumor tissue, while reducing the expression of IL-10 and IL13 factors [19]. On the other hand, IL-17A was reported to promote lung cancer by increasing cytokines such as IL6, granulocyte-colony stimulating factor (G-CSF), Milk fat globule epidermal growth factor 8 (MFG-E8), and the chemokine ligand 1 (CXCL1) in a murine model [21]. In this model, tumor-associated neutrophils were significantly increased, and lymphocyte recruitment was significantly reduced. As a reported therapeutic model, blocking IL-17A improved the efficacy of PD-1 Ab therapy in colorectal cancer in mice [20].

Our initial attempt was to reevaluate the role of IL-17 within three types of murine tumor models. We have tested the function of IL-17A Ab combined with the established immune cancer therapies.

At first, we tested the effectiveness of IL-17A Ab in addition to the immune checkpoint inhibitor, PD-1 Ab, in murine cancer models. Our experimental data demonstrated that the combination of IL-17A Ab and PD-1 Ab had the best performance for tumor suppression in the murine melanoma implant model. In further examination of the mechanism underlying combination therapy, bioinformatics analysis of the tumor samples indicated enhanced infiltration of activated CD4+ T cells and CD8+ T cells. Activated CD4+ or CD8+ T cells were detected in lymph nodes as well. The effectiveness of IL-17A Ab plus PD-1 Ab was also proven in the UVB-induced skin carcinogenesis model in mice. UVB-induced skin cancer development was significantly delayed after the combination treatment with IL-17A Ab and PD-1 Ab. Combinational strategies with CAR-T cell therapy have been reported in clinical studies targeting CD19 or B cell maturation antigen (BCMA) [4]. In our study, the advantage of adding IL-17A Ab was also proved in T cell therapy against B16-OVA. Significant tumor suppression was observed with the combination of IL-17A Ab plus OVA-specific T cells in the B16-OVA model compared with solely OVA-specific T cells.

Thus, cancer immunotherapies with IL-17 Ab have shown promising efficacy. However, a few reports warned of possible immune escape after blocking the IL-17A pathway, which may lead to tumor relapse [8]. Here, our second goal was to confirm the use of IL-17A Ab application without any adverse effects. There was no adverse effect observed with IL-17A Ab application in either solo usage or in combination with PD-1 Ab in the tested models, and in the UVB-induced skin carcinogenesis model in mice. In addition, there were no adverse effects of IL-17A Ab treatments in T cell therapy against B16-OVA melanoma. Our data have demonstrated that the application of IL-17A Ab either solo or in combination with current tumor immunotherapy was effective and did not show any negative effects on tumor relapse, as reported previously [8]. Our data suggest that the use of anti-IL-17 antibodies may not be detrimental if the checkpoint inhibitor treatment continues.

CRS is known to be a major adverse effect in tumor immunotherapies. Currently, anti-IL6 Ab, glucocorticoids, anti-IL1, and several other reagents have been used for CRS in cancer immunotherapies [4,22]. Our findings may encourage anti-IL-17 Ab as a strong candidate for CRS treatment in future settings.

Our dataset had limitations. Long-term assessment was not feasible due to the rapid progression of implanted tumors without treatment. Additionally, our study consisted of simultaneous administration of IL-17A and PD-1 antibodies, and we did not analyze the effects of their sequential administration. We have shown evidence of efficacy by adding IL-17A Ab to the current cancer immunotherapy in a melanoma model. Thus, our findings may contribute to the future expansion of human cancer immunotherapy regimens.

## Data Availability

The original contributions presented in this study are included in the article. Further inquiries can be directed to the corresponding author.
